# Mediterranean diet during pregnancy and childhood respiratory and atopic outcomes: birth cohort study

**DOI:** 10.1183/13993003.01215-2019

**Published:** 2020-03-12

**Authors:** Annabelle Bédard, Kate Northstone, A. John Henderson, Seif O. Shaheen

**Affiliations:** 1 Centre for Primary Care and Public Health, Barts and The London School of Medicine and Dentistry, Queen Mary University of London, London, UK; 2 Population Health Sciences, Bristol Medical School, University of Bristol, Bristol, UK

## Abstract

Evidence for associations between Mediterranean diet during pregnancy and childhood asthma, allergy and related outcomes is conflicting. Few cohorts have followed children to school age, and none have considered lung function.

In the Avon Longitudinal Study of Parents and Children, we analysed associations between maternal Mediterranean diet score during pregnancy (estimated by a food frequency questionnaire, using an *a priori* defined score adapted to pregnant women; score ranging from 0 (low adherence) to 7 (high adherence)) and current doctor-diagnosed asthma, wheeze, eczema, hay fever, atopy and lung function in 8907 children at 7–9 years. Interaction between maternal Mediterranean diet and maternal smoking in pregnancy was investigated.

The maternal Mediterranean diet score was not associated with asthma or other allergic outcomes. Weak positive associations were found between maternal Mediterranean diet score and childhood maximal mid-expiratory flow (forced expiratory flow at 25–75% of forced vital capacity (FEF_25–75%_)) after controlling for confounders. Higher Mediterranean diet scores were associated with increased FEF_25–75%_ z-scores adjusted for age, height and sex (β 0.06, 95% CI 0.01–0.12; p=0.03, comparing a score of 4–7 *versus* a score of 0–3). Stratifying associations by maternal smoking during pregnancy showed that associations with FEF_25–75%_ were only seen in children of never-/passive-smoking mothers, but no evidence for a statistically significant interaction was found.

Results suggest adherence to a Mediterranean diet during pregnancy may be associated with increased small airway function in childhood, but we found no evidence for a reduced risk of asthma or other allergic outcomes.

## Abstract 2

**Adherence to a Mediterranean diet during pregnancy is not associated with a reduced risk of asthma or other allergic outcomes in the offspring, but may be associated with increased small airway function in childhood** http://bit.ly/382yGcF

## Introduction

A Mediterranean diet is typified by a high intake of vegetables, legumes, fruits and nuts, unrefined cereals, fish and olive oil, a low-to-moderate intake of dairy products and a low intake of meat and poultry and saturated fats [1]. In a Dutch birth cohort study, low adherence to a Mediterranean-like diet in pregnancy was associated with lower birthweight [2], and a recent randomised controlled trial conducted in pregnant women found that a Mediterranean diet intervention (with additional extra virgin olive oil and pistachio nuts) reduced the rate of small-for-gestational-age newborns, prematurity and gestational weight gain [3]. It also reduced gestational diabetes [3], which has been associated with an increased risk of atopic eczema and atopy in one birth-cohort study conducted in the United States [4]. Given that low birthweight, prematurity and gestational weight gain are risk factors for childhood asthma [5–7], and low birthweight and premature birth are also risk factors for lower childhood lung function [8, 9], high adherence to a Mediterranean diet in pregnancy might be expected to protect against asthma and/or impaired lung function in the offspring. A number of birth-cohort [10–12] and retrospective [13] studies have investigated whether adherence to a Mediterranean diet in pregnancy might protect against the development of asthma and allergies in the offspring, and a recent systematic review concluded that there was some evidence that higher adherence was associated with a lower risk of wheezing in infancy, but evidence for a lower risk of asthma, wheezing and atopic outcomes later in childhood was lacking [14]. Only one prospective study, conducted in Menorca, followed the children to school age [10] and none examined associations with lung function. As a Mediterranean diet is rich in antioxidants, we might expect any beneficial effects of high adherence to a Mediterranean diet on childhood outcomes to be greatest among offspring of mothers who smoked in pregnancy, since tobacco smoke is a source of oxidative stress [15], and that high adherence to a Mediterranean diet would attenuate the detrimental effects of maternal smoking.

In a large UK population-based birth cohort, we have investigated whether greater adherence to a Mediterranean diet in pregnancy is associated with a reduced risk of asthma and other atopic outcomes and with higher lung function in the offspring at school age.

## Methods

### Participants

The Avon Longitudinal Study of Parents and Children (ALSPAC) is a population-based birth cohort which recruited 14 541 predominantly white pregnant women resident in Avon, UK with expected dates of delivery April 1, 1991 to December 31, 1992. These pregnancies resulted in 13 972 singleton or twin children who were alive at 1 year of age. The cohort has been followed since birth with annual questionnaires and, since age 7 years, with objective measures in annual research clinics. The study protocol has been described previously [16, 17]. The study website contains details of all the data that are available through a fully searchable data dictionary and variable search tool: www.bristol.ac.uk/alspac/researchers/our-data/. Ethics approval was obtained from the ALSPAC ethics and law committee (IRB 00003312) and the local National Health Service research ethics committees. Informed consent for the use of data collected *via* questionnaires and clinics was obtained from participants following the recommendations of the ALSPAC ethics and law committee at the time.

### Exposure assessment

Data on maternal diet in pregnancy were collected by a food frequency questionnaire (FFQ) at 32 weeks’ gestation, covering all the main foods consumed in Britain at the time [18]. This FFQ was based on the one used by Yarnell *et al.* [19] in a British population, which has been validated against weighed dietary records, and modified in the light of a more recent weighed dietary survey [18]. It has been shown to produce mean nutrient intakes for the mothers [18] that were similar to those obtained for women in the British National Diet and Nutritional survey for adults [20, 21].

The questionnaire asked about current weekly frequency of consumption of 43 food groups and food items, with the possibility for respondents to tick one of the following options: never or rarely, once in 2 weeks, 1–3 times a week, 4–7 times a week, more than once a day.

More detailed questions were asked about daily consumption of a further eight basic foods (including the number of spoons of sugar, cups of coffee and cups of tea, per day). The FFQ was used to estimate total energy intake and daily nutrient intake, by multiplying the daily frequency of consumption of a food by the nutrient content [22] of a standard portion [23] of that food, and summing this for all the foods consumed. Information on portion size was not collected. In order to apply quantitative meaning to the frequency categories, the data were numerically transformed into times per week as follows: 0, 0.5, 2, 5.5 and 10 times per week. The number of slices of bread consumed each day on average was recorded separately and the amount of milk consumed was estimated from the number of cups of white tea/coffee consumed per day and the frequency of breakfast cereal, milky puddings and milk drinks consumed per week.

A Mediterranean diet score was based on that devised by Chatzi *et al*. [10] for pregnant women, which was adapted from the original Mediterranean diet score by Trichopoulou *et al*. [1]. Chatzi *et al.* included dairy products as beneficial, rather than detrimental, and did not include alcohol. The score is based on the median weekly intake of six beneficial food groups (vegetables, legumes, fruits and nuts, cereal, fish and dairy) and one detrimental food group (meat) (supplementary table S1 presents a detailed description of the food groups from the FFQ contributing to each component of the score, and the median weekly consumption in ALSPAC women). Women whose consumption of the beneficial food groups was above the median were assigned a value of 1, and those below were assigned a value of 0. Conversely, for the detrimental food group, consumption below the median was assigned a value of 1, and above the median was assigned a value of 0. The seven food group values were then summed together to obtain a score which ranged from 0 to 7, with a higher score representing greater adherence to a Mediterranean-style diet.

### Outcome assessment

Current doctor-diagnosed asthma was defined in children at 7.5 years (primary outcome) if mothers responded positively to the question “Has a doctor ever actually said that your study child has asthma?” and to one or both of the questions “Has your child had any of the following in the past 12 months: wheezing with whistling; asthma?”.

Current wheezing, eczema and hay fever in children at 7.5 years were defined by a positive answer to the question: “Has your child had any of the following in the past 12 months: wheezing with whistling; eczema; hay fever?”.

Atopy at 7 years was defined as a positive reaction (maximum diameter of any detectable weal) to *Dermatophagoides pteronyssinus*, cat or grass (after subtracting positive saline reactions from histamine and allergen weals, and excluding children unreactive to 1% histamine).

Lung function was measured by spirometry (Vitalograph 2120; Vitalograph, Buckingham, UK) at age 8.5 years after withholding short-acting bronchodilators for ≥6 h and long-acting bronchodilators and theophyllines for ≥24 h. The best of three reproducible flow–volume curves was used to measure forced expiratory volume in 1 s (FEV_1_), forced vital capacity (FVC) and maximal mid-expiratory flow (forced expiratory flow at 25–75% of FVC (FEF_25–75%_)), which were further transformed to age-, height- and sex-adjusted standard deviation units [24]. The tests adhered to American Thoracic Society (ATS) criteria for standardisation and reproducibility of flow–volume measurement [25], with the exception of ATS recommendations for duration of expiration [26]; as many children did not fulfil forced expiratory time >6 s end-of-test criteria, a minimal volume change over the final 1 s was used. Additionally, lung function was measured at 15 years in 3549 ALSPAC children.

### Potential confounders

We selected potential confounding factors which are known (from existing literature) to be associated with one or more of the outcomes of interest [27, 28]. These included maternal age at delivery, sex of child, multiple pregnancy, season of birth, maternal history of atopic diseases (hay fever, asthma, eczema, allergies or attacks of wheezing with whistling on the chest or attacks of breathlessness in the past 2 years), parity, highest educational qualification in UK schools (Certificate of Secondary Education, vocational, Ordinary level, Advanced level, degree), housing tenure, financial difficulties, ethnicity, breastfeeding duration and maternal factors during pregnancy (smoking status, anxiety score (Crown–Crisp Experiential Index) [29], paracetamol use, antibiotic use, infections (urinary infection, influenza, rubella, thrush, genital herpes, other), supplement use (iron, zinc, calcium, folic acid, vitamins, “others”) and total energy intake (kJ·day^−1^)). Smoking status was categorised as the maximum exposure during pregnancy (never, passive smoking only, 1–9 cigarettes·day^−1^, 10–19 cigarettes·day^−1^, ≥20 cigarettes·day^−1^).

### Statistical analyses

We compared the distributions of child and maternal variables across the maternal Mediterranean diet score categories (≥4 *versus* ≤3, as done previously by Chatzi *et al*. [10]) using t-tests for differences in continuous variables and Chi-squared tests for differences in categorical variables. Logistic regression, multinomial logistic regression and linear regression were used to analyse relations between the maternal Mediterranean diet score in pregnancy and binary, categorical and continuous outcomes, respectively. We analysed the maternal Mediterranean diet score first as a binary variable (*i.e.* ≥4 and ≤3 [10]) using the lower category as reference, and second as a continuous variable to test for linear trend (*i.e.* per increasing score-unit effect). For all regression analyses, two stages of adjustment were used. In model 1 we adjusted for total energy intake only. In model 2 we adjusted additionally for all potential confounders listed above.

When evidence for associations persisted, we considered other factors which could be considered as potential mediators of associations between maternal Mediterranean diet in pregnancy and childhood outcomes, namely, prematurity [3, 7, 9], impaired fetal growth [2, 3, 5, 8], maternal obesity (which can be viewed either as confounder or mediator) and weight gain [3, 6] and offspring obesity [30, 31]. Therefore, we adjusted additionally for maternal pre-pregnancy body mass index (BMI) (self-reported, categorised according to World Health Organization categories [32]), gestational age at delivery, birthweight (<2500, 2500–2999, 3000–3499, 3500–3999, ≥4000 g [5, 33]), maternal weight gain during pregnancy (categorised in quartiles) (all abstracted from obstetric records) and child's BMI at 7 years (based on measured height and weight at clinic, categorised into <15.00, 15.00–17.49, 17.50–20.49, ≥20.50 kg·m^−2^ [33]), using separate models (*i.e.* one model for each potential mediator), to investigate potential mediation (supplementary figure S1 shows a directed acyclic graph). As a Mediterranean diet is rich in antioxidants, we might expect any beneficial effects on childhood outcomes to be greatest among offspring of mothers who smoked in pregnancy, since tobacco smoke is a source of oxidative stress [15]. We therefore stratified the dietary analyses by maternal smoking history (dichotomised) to explore potential effect modification by smoking and tested for interaction. In addition, we explored whether the association between maternal smoking in pregnancy and FEF_25–75%_, previously reported in ALSPAC [34], was modified by Mediterranean diet score (dichotomised).

As sensitivity analyses, we repeated analyses after exclusion of mothers with implausible energy intakes, defined as total daily energy intake below the bottom 5% (*i.e.* 1028 kcal·day^−1^) or above the top 5% (*i.e.* 2560 kcal·day^−1^). To correct for potential loss-to-follow-up bias, we used inverse probability weighting and assigned to each woman a weight that is the inverse of the probability of her selection for given values of covariates (further details are given in the supplementary material) [35]. To investigate potential nonlinear effects, we repeated our analyses of the associations between the maternal Mediterranean diet score and childhood outcomes, first considering the Mediterranean diet score in two categories, comparing women with a score of 6–7 to women with a score 0–5, and second considering the Mediterranean diet score in four categories (0–1 (reference category), 2–3, 4–5 and 6–7).

All statistical analyses were performed using Stata (version 12.1; StataCorp, College Station, TX, USA).

## Results

Of the 13 972 singleton or twin children alive at 1 year of age, information on maternal diet was available for 11 993, of whom there was information on at least one of the outcomes of interest for 8907 (supplementary figure S2). Characteristics of the 8907 mother–child pairs included in the analyses, and those of the 3086 mother–child pairs with information on maternal diet who were excluded because of missing outcome data at 7–9 years (*i.e.* lost to follow-up), are compared in supplementary table S2.

Women in the higher category of Mediterranean diet score during pregnancy were older and more educated than women in the lower category. They were more likely to give birth in winter or spring, to breastfeed for ≥3 months, to have an owned/mortgaged house and to use supplements during pregnancy. They were less likely to have financial difficulties, to have a high anxiety score, to smoke or to use paracetamol during pregnancy. In addition, they had a lower pre-pregnancy BMI, higher total energy intake and gained more weight during pregnancy. Their offspring were more likely to have weighed more at birth and less likely to have a high BMI at 7 years (table 1).

**TABLE 1:** Characteristics of mothers and offspring who had information on at least one of the outcomes of interest (wheeze, asthma, atopy, eczema, hayfever, total IgE, lung function) by maternal Mediterranean diet score in pregnancy (n=8907)

	**Mediterranean diet score**		**p-value^#^**
	**0–3**	**4–7**	
**Subjects n**	3475	5432	
**Mother's age years**	28.1±4.7	29.4±4.5	<0.001
**Parity (living children)**			
0	45.2	45.7	0.83
1	36.3	35.7	
≥2	18.5	18.6	
**Sex of child**			
Male	50.0	51.9	0.08
Female	50.0	48.1	
**Multiple pregnancy**			
Singleton	97.5	97.6	0.66
Twin	2.5	2.4	
**Season of birth**			
Winter	15.9	16.3	<0.001
Spring	25.1	28.3	
Summer	30.0	30.1	
Autumn	29.0	25.4	
**Breastfeeding duration**			
Never	28.6	16.5	<0.001
<3 months	35.3	29.2	
3–6 months	12.2	14.8	
≥6 months	23.9	39.5	
**Mother's educational level**			
Certificate of Secondary Education	21.3	11.6	<0.001
Vocational	11.7	7.3	
Ordinary level	38.7	33.4	
Advanced level	19.4	28.7	
Degree	8.9	19.0	
**Maternal ethnicity**			
White	98.0	98.3	0.38
Non-white	2.0	1.7	
**Housing tenure**			
Owned/mortgaged	78.2	87.3	<0.001
Council rented	14.2	6.4	
Non-council rented	7.6	6.3	
**Financial difficulties**			
Yes	19.8	15.4	<0.001
**Maternal history of atopic diseases**			
Yes	69.1	67.9	0.24
**Maternal anxiety score in pregnancy**			
0–9	18.4	23.1	<0.001
10–14	25.0	26.2	
15–20	25.7	26.1	
≥20	31.0	24.7	
**Maximum maternal tobacco exposure**			
None	21.5	29.7	<0.001
Passive only	44.7	46.7	
1–9 cigarette·day^−1^	7.9	7.9	
10–19 cigarette·day^−1^	14.1	9.6	
≥20 cigarette·day^−1^	11.8	6.0	
**Maternal paracetamol use during pregnancy**			
Yes	64.6	61.0	0.001
**Maternal antibiotic use during pregnancy**			
Yes	16.0	16.2	0.81
**Maternal dietary supplement use during pregnancy**			
Yes	54.4	58.4	<0.001
**Maternal infections in pregnancy**			
Yes	45.1	46.2	0.33
**Total energy intake kcal·day^−1^**	1600±451	1826±459	<0.001
**Maternal pre-pregnancy BMI kg·m^−2^**			
<18.50	4.1	4.4	<0.001
18.50–24.99	71.4	77.8	
25.00–29.99	17.4	13.7	
≥30.00	7.1	4.1	
**Birthweight g**			
<2500	5.0	3.8	0.006
2500–2999	14.6	13.3	
3000–3499	35.8	35.3	
3500–3999	31.9	34.0	
≥4000	12.7	13.7	
**Gestational age weeks**	39.4±1.8	39.5±1.8	0.13
**Child's BMI at 7** **years kg·m^−2^**			
<15.00	28.0	28.1	0.01
15.00–17.49	51.7	53.0	
17.50–20.49	15.2	15.2	
≥20.50	5.2	3.6	
**Maternal weight gain during pregnancy**			
Quartile 1 (<9.7 kg)	27.8	23.7	0.001
Quartile 2 (9.7–12.5 kg)	23.8	25.5	
Quartile 3 (12.5–15.5 kg)	25.1	25.7	
Quartile 4 (≥15.5 kg)	23.4	25.1	

Data are presented as mean±sd or %, unless otherwise stated. Ig: immunoglobulin; BMI: body mass index. ^#^: Chi-squared tests were used for categorical variables, and t-tests were used for continuous variables.

After controlling for confounders, maternal Mediterranean diet score (whether analysed as a binary or continuous variable), was not associated with asthma, wheeze, eczema, hayfever or atopy (table 2). When we analysed the associations between the maternal Mediterranean diet score and childhood lung function at 8–9 years after controlling for total energy intake only, strong evidence was found for positive associations with childhood FEV_1_ and FEF_25–75%_. These associations slightly weakened after further adjustment for potential confounders, but evidence for a positive association with childhood FEF_25–75%_ persisted when comparing higher *versus* lower maternal Mediterranean diet score (difference in age-, height- and sex-adjusted standard deviation units 0.06, 95% CI 0.01–0.12; p-value 0.03) (table 3). When we analysed the relationship between Mediterranean diet score and childhood lung function at 15 years, the per-unit increase effect estimates for FEF_25–75%_ were similar to those observed at 8 years (supplementary table S3); however, given the smaller sample size, associations were no longer conventionally significant.

**TABLE 2:** Associations between maternal Mediterranean diet score during pregnancy and asthma, wheeze, eczema, hay fever and atopy in the offspring (n=8629)

	**Subjects**	**Mediterranean diet score**			
		**4–7 *versus* 0–3**	**p*-*value**	**Per-unit increase**	**p*-*trend**
**Asthma**	7634				
Model 1^#^		0.94 (0.81–1.09)	0.41	0.97 (0.92–1.01)	0.15
Model 2^¶^		1.03 (0.88–1.20)	0.71	1.00 (0.95–1.05)	0.93
**Wheeze**	7719				
Model 1^#^		1.02 (0.88–1.19)	0.80	1.01 (0.96–1.06)	0.84
Model 2^¶^		1.04 (0.89–1.22)	0.62	1.01 (0.96–1.07)	0.63
**Eczema**	7705				
Model 1^#^		1.13 (0.99–1.29)	0.07	1.03 (0.99–1.07)	0.18
Model 2^¶^		1.10 (0.96–1.26)	0.18	1.01 (0.97–1.06)	0.55
**Hay fever**	7685				
Model 1^#^		1.00 (0.85–1.18)	0.99	1.01 (0.96–1.07)	0.72
Model 2^¶^		0.97 (0.81–1.15)	0.69	1.00 (0.94–1.06)	0.97
**Atopy**	6078				
Model 1^#^		1.03 (0.90–1.17)	0.66	1.03 (0.98–1.07)	0.23
Model 2^¶^		0.94 (0.82–1.07)	0.34	0.99 (0.95–1.04)	0.81

Data are presented as n or OR (95% CI), unless otherwise stated. ^#^: controlling for energy intake; ^¶^: controlling for energy intake, smoking, infections, supplements, antibiotics and paracetamol use during pregnancy; maternal educational level, housing tenure, financial difficulties, ethnicity, age, parity, history of atopic diseases, anxiety; sex of child, season of birth, multiple pregnancy, breastfeeding duration.

**TABLE 3:** Associations between maternal Mediterranean diet score during pregnancy (binary and continuous) and FEV_1_, FVC and FEF_25–75%_ in the offspring (n=6120)

	**Subjects n**	**Mediterranean diet score**			
		**4–7 *versus* 0–3**	**p*-*value**	**Per unit increase**	**p*-*trend**
**FEV_1_**	6026				
Model 1^#^		0.07 (0.01–0.12)	0.01	0.03 (0.01–0.04)	0.005
Model 2^¶^		0.05 (−0.01–0.10)	0.11	0.02 (0.00–0.04)	0.06
**FVC**	6120				
Model 1^#^		0.03 (−0.03–0.08)	0.36	0.01 (0.00–0.03)	0.12
Model 2^¶^		0.01 (−0.05–0.06)	0.78	0.01 (−0.01–0.03)	0.38
**FEF_25–75%_**	6120				
Model 1^#^		0.08 (0.02–0.13)	0.005	0.02 (0.01–0.04)	0.01
Model 2^¶^		0.06 (0.01–0.12)	0.03	0.02 (0.00–0.04)	0.06

Data are presented as n or β (95% CI), unless otherwise stated. FEV_1_: forced expiratory volume in 1 s; FVC: forced vital capacity; FEF_25–75%_: maximal mid-expiratory flow (forced expiratory flow at 25–75% of FVC); β: difference in age-, height- and sex-adjusted standard deviation units. ^#^: controlling for energy intake; ^¶^: controlling for energy intake, smoking, infections, supplements, antibiotics and paracetamol use during pregnancy; maternal educational level, housing tenure, financial difficulties, ethnicity, age, parity, maternal history of atopic diseases, anxiety score; sex of child, season of birth, multiple pregnancy, breastfeeding duration.

Additional separate adjustment for maternal pre-pregnancy BMI, gestational age at delivery, birthweight, maternal weight gain during pregnancy and child's BMI at 7 years did not alter the main findings (data not shown), and therefore no further formal mediation analysis was conducted. Excluding mothers with implausible energy intakes did not alter the main findings, nor did the inverse probability weighting analysis (data not shown).

When we stratified the analyses of Mediterranean diet and lung function by maternal smoking in pregnancy, maternal Mediterranean diet was positively associated with childhood FEF_25–75%_ among non-/passive smokers, but not among active smokers, but no evidence for a statistically significant interaction was found (table 4). Conversely, when we stratified the negative association between maternal smoking in pregnancy and FEF_25–75%_ by Mediterranean diet score (dichotomised), there was no evidence of attenuation of the association by higher adherence to a Mediterranean diet (data not shown).

**TABLE 4:** Associations between maternal Mediterranean diet score during pregnancy and childhood lung function stratified by maternal smoking during pregnancy (n=6115)

	**Subjects**	**Mediterranean diet score β^#^ (95% CI)**			
		**4–7 *versus* 0–3**	**p*-*value**	**Per unit increase**	**p*-*trend**
**FEV_1_**					
Non-/passive smokers	4479	0.05 (−0.02–0.11)	0.15	0.02 (0.00–0.04)	0.10
Active smokers	1542	0.04 (−0.07–0.15)	0.45	0.02 (−0.02–0.06)	0.29
p interaction^¶^		0.81		0.96	
**FVC**					
Non-/passive smokers	4558	0.01 (−0.05–0.08)	0.71	0.01 (−0.02–0.03)	0.59
Active smokers	1557	0.01 (−0.09–0.12)	0.80	0.02 (−0.02–0.05)	0.33
p interaction^¶^		0.69		0.90	
**FEF_25–75%_**					
Non-/passive smokers	4558	0.07 (0.00–0.13)	0.04	0.02 (0.00–0.04)	0.05
Active smokers	1557	0.04 (−0.07–0.15)	0.44	0.01 (−0.03–0.04)	0.70
p interaction^¶^		0.86		0.68	

β: difference in age-, height- and sex-adjusted standard deviation units; FEV_1_: forced expiratory volume in 1 s; FVC: forced vital capacity; FEF_25–75%_: maximal mid-expiratory flow (forced expiratory flow at 25–75% of FVC). ^#^: controlling for energy intake, infections, supplements, antibiotics and paracetamol use during pregnancy; maternal educational level, housing tenure, financial difficulties, ethnicity, age, parity, maternal history of atopic diseases, anxiety score; sex of child, season of birth, multiple pregnancy, breastfeeding duration; ^¶^: treating smoking as a binary variable and the Mediterranean diet score as either a binary or continuous variable.

When analyses of the associations between the maternal Mediterranean diet score and childhood FEV_1_ and FEF_25–75%_ were repeated, considering the Mediterranean diet score in two categories (0–5 (reference category) and 6–7) or in four categories (0–1 (reference category), 2–3, 4–5 and 6–7) to investigate the linearity of the association, results were consistent with linear trends (data not shown).

## Discussion

This is the largest observational study to investigate the relationship between Mediterranean diet in pregnancy and childhood respiratory and allergic outcomes. A limitation of the evidence to date is that many studies have assessed maternal diet retrospectively or have only investigated outcomes in infancy. Of two cohort studies which assessed Mediterranean diet during pregnancy and outcomes beyond infancy, one small study from Menorca (n=460), using the same maternal Mediterranean diet score, reported significant negative associations with wheeze and atopy at 6.5 years [10], but another, larger, study from the US, using a slightly different score (n=1376), found no association with asthma, wheeze or atopy at 3 years [12]. Given the much larger size of ALSPAC, and our null findings for childhood asthma, wheeze and allergic outcomes, we would argue that the weight of current prospective evidence suggests that adherence to a Mediterranean diet in pregnancy is unlikely to reduce the risk of these conditions at school age. However, we found weak evidence that a higher Mediterranean diet score during pregnancy was associated with higher FEF_25–75%_ in the offspring, after controlling for potential confounders. Previous studies have not investigated the relationship between Mediterranean diet in pregnancy and childhood lung function; to the best of our knowledge this is a novel finding.

### Mechanisms

If the association between Mediterranean diet in pregnancy and lung function in the offspring is causal, one plausible explanation is that it is mediated through the high antioxidant content of the fruit, vegetables and cereals in a Mediterranean diet [14]. If this were the case, we would have expected to see an interaction between maternal Mediterranean diet score in pregnancy and maternal smoking on childhood FEF_25–75%_. To our knowledge, this has not been investigated before. We hypothesised, *a priori*, that a higher Mediterranean diet score in pregnancy might be particularly beneficial if the fetus was exposed to tobacco smoke, by protecting the developing lung from potentially damaging oxidative stress [15]. In fact, there was no association between a Mediterranean diet in pregnancy and lung function among active smokers. On the contrary, an association was only seen among mothers who had not actively smoked in pregnancy. An alternative, *post hoc* hypothesis could be that benefits are only seen above a certain threshold of adherence to a Mediterranean diet, and we have confirmed that mothers who did not actively smoke in pregnancy had a higher Mediterranean diet score than those who did. Additionally, we found no evidence that the detrimental effects of maternal smoking on childhood FEF_25–75%_ were attenuated by higher adherence to a Mediterranean diet. Apart from its antioxidant properties, a Mediterranean diet may have anti-inflammatory effects [36]. Part of this effect may reflect the anti-inflammatory properties of omega-3 polyunsaturated fatty acids in oily fish. We speculate that this might partly explain the association between a Mediterranean diet in pregnancy and offspring lung function that we observed; a recent trial reported that fish oil derived omega-3 fatty acid supplementation in pregnancy reduced the risk of early childhood wheezing in the offspring [37], and early childhood wheezing is associated with later reductions in lung function [38]. We found no evidence to suggest that the association between maternal Mediterranean diet score and childhood FEF_25–75%_ was mediated by maternal BMI, gestational weight gain or child's BMI, nor by prematurity or low birthweight.

### Strengths and limitations

Strengths of the ALSPAC birth cohort include its population-based prospective design, rich information on numerous potential confounders, detailed phenotypic outcome measurements, and its size, which gave us greater statistical power than the previous birth cohort study that has investigated this research question in offspring of school age [10].

The Mediterranean diet score has been developed in Mediterranean countries and is based on population-specific median values. Thus, it may not be adapted to non-Mediterranean countries such as the UK, in which median intakes of some specific foods may be lower, and potential beneficial effects might be missed. We acknowledge that this might be an explanation for why an association was found between the maternal Mediterranean diet and childhood asthma in children from Menorca (*i.e.* a Mediterranean population) [10] and not in children in the USA [12] or in ALSPAC children. However, the fact that similar results were observed in our data when the Mediterranean diet score was studied as a binary (above *versus* below median, highest categories *versus* low or medium score) or as a continuous variable makes this possibility less likely. A previous study of maternal dietary patterns in pregnancy in relation to childhood respiratory outcomes has been conducted in ALSPAC, using principal component analysis (PCA) to derive dietary patterns [39]. That study showed that dietary patterns in pregnancy, including a “health-conscious” pattern (which had some similarities to a Mediterranean diet), did not predict asthma and related outcomes in the offspring after controlling for confounders. However, data-driven methods such as PCA are population-specific, and using *a priori* approaches such as the Mediterranean diet score is more relevant in terms of public health. Other *a priori* approaches such as the Alternate Healthy Eating Index (AHEI) score, which is based on international guidelines and has been adapted for pregnant women (AHEI-P) [40], may be more adapted to non-Mediterranean populations; however, given the lack of information on some specific AHEI-P food/nutrient items in ALSPAC's FFQ, *e.g*. *trans*-fat and whole grains (although partly covered by cereals), it was not applicable in ALSPAC pregnant women.

Although the FFQ that we used had not been formally calibrated against other instruments such as diet diaries, it was based on the one used by Yarnell *et al.* [19] which has been validated against weighed dietary records, and modified in the light of a more recent weighed dietary survey [18]. Although the limitations of the FFQ method are well known, reproducibility and validity of FFQs have been studied and found to be relatively good overall [41]. While there might have been some misclassification of dietary exposures (*e.g*. the ALSPAC FFQ did not allow us to distinguish between white and wholegrain bread, between white and brown rice and between red and processed meat; and bread consumption was assessed differently compared to other food groups, and thus may have been overestimated), this is likely to have been non-differential with respect to the outcomes of interest, and would be expected to bias effect estimates towards the null; in other words, the magnitude of associations may have been underestimated, and small or modest effects may have been missed. The possibility that the association between Mediterranean diet in pregnancy and offspring lung function might be explained by uncontrolled or residual confounding cannot be ruled out, especially given that the Mediterranean diet score is highly correlated with social and lifestyle factors. However, we think that this is unlikely, as we controlled for numerous potential confounders in the analyses. Another limitation is that child's Mediterranean diet score was not available in ALSPAC and hence we cannot rule out potential confounding by postnatal diet. However, since most findings are null, childhood diet would have to be acting as a negative confounder for significant effects of maternal Mediterranean diet to appear on adjustment, which seems unlikely. As with any longitudinal study, we cannot rule out the possibility that exclusion of mother–child pairs without complete information might have biased our findings. However, it could be argued that, for our results for the Mediterranean diet score and childhood lung function to be totally spurious in those included in our analysis (and for the associations to be truly null in the population as a whole), associations in the excluded mother–child pairs would have to be at least of equal magnitude in the opposite direction, which seems unlikely. Furthermore, loss to follow-up bias has been shown to only slightly modify associations in longitudinal studies, including in ALSPAC [42], and the results of our inverse probability weighting analysis confirmed that loss to follow-up is unlikely to have biased our results. In view of the multiple analyses undertaken, we cannot exclude the possibility that the associations between Mediterranean diet in pregnancy and offspring lung function occurred by chance; hence they should be interpreted with caution and require replication in another birth cohort study. Given the *a priori* nature of the general hypothesis being tested (*i.e*. a beneficial effect of a Mediterranean diet), and the fact that some outcomes of interest were highly correlated, it did not seem appropriate to correct for multiple testing.

### Conclusions

We found weak evidence that greater adherence to a Mediterranean diet in pregnancy is associated with higher small airway function in the offspring, but is not associated with a reduced risk of asthma or other allergic outcomes. Further studies in school-aged children are needed to confirm these results.

## Supplementary material

10.1183/13993003.01215-2019.Supp1 **Please note:** supplementary material is not edited by the Editorial Office, and is uploaded as it has been supplied by the author.
Supplementary material ERJ-01215-2019.SUPPLEMENT

## Shareable PDF

10.1183/13993003.01215-2019.Shareable1 This one-page PDF can be shared freely online.
Shareable PDF ERJ-01215-2019.Shareable
