# Supplementary material for: Mediterranean diet during pregnancy and childhood respiratory and atopic outcomes: birth cohort study
Source: Eur Respir J. 2020 Mar 12;55(3):1901215. doi: 10.1183/13993003.01215-2019 (PMC7066469; doi:10.1183/13993003.01215-2019)
Supplement: Supplementary file 1 [file ERJ-01215-2019.SUPPLEMENT.pdf]

Online data supplement

Mediterranean diet during pregnancy and childhood respiratory and atopic outcomes: birth cohort study

Annabelle Bédard[1], PhD, Kate Northstone[2], PhD, PhD, A John Henderson[2*], MD, Seif O Shaheen[1*], PhD

[1]Centre for Primary Care and Public Health, Barts and The London School of Medicine and Dentistry, London, Queen Mary University of London, UK

[2] Population Health Sciences, Bristol Medical School, University of Bristol, UK

[*] Joint senior authors

Corresponding author:

Prof. Seif Shaheen, Centre for Primary Care and Public Health, Blizard Institute, Barts and The London School of Medicine and Dentistry, 58 Turner Street, London E1 2AB.

Email: s.shaheen@qmul.ac.uk

Tel: +44 (0)20 7882 2480; Fax: +44 (0)20 7882 2252

Supplementary methods

Inverse probability weighting

Inverse probability weighting has been proposed as a way to correct for selection bias [1]. By assigning to each subject a weight that is the inverse of the probability of his/her selection based on a given set of covariates and exposure, inverse probability weighting creates a pseudo-population in which effect measures are not affected by selection bias (provided that the outcome in the uncensored subjects truly represents the outcome in the censored subjects for the same values of covariates and exposure). We used this approach by estimating for each woman, the probability of her selection for given values of covariates (ie. the characteristics for which differences between excluded and included women were found to be statistically significant, including the exposure – see online Table 1) and assigning her a weight that is the inverse of that probability.

Online Table S1: Food groups from the FFQ contributing to the Mediterranean score calculation and estimated median weekly consumption.

MD Components	**Original Med Score** (Trichopoulou et al. *BMJ* 1995[2]) **Criterion for scoring 1***	**Med Score adapted to pregnancy** (Chatzi et al. Thorax 2008[3]) used in the present study **Criterion for scoring 1***	**Food groups from FFQ**[1]	**Median weekly consumption in ALSPAC women**	**% of mother scoring 1**
**Beneficial food groups**					
**Vegetables** (servings/week)	≥ median	≥ median	Peas/sweetcorn<br>Green leafy vegetables<br>Other green vegetables<br>Carrots<br>Other root vegetables<br>Salad	10.5 servings/ week	50.9%
**Legumes** (servings/ week)	≥ median	≥ median	Baked beans<br>Pulses<br>Bean curd<br>Tahini<br>Soya meat	2.0 servings/ week	60.2%
**Fruits and nuts** (servings/ week)	≥ median	≥ median	Fresh fruit<br>Nuts	5.5 servings/ week	66.4%
**Cereals** (servings/ week)	≥ median	≥ median	Rice<br>Oat cereals<br>Bran cereals<br>Other cereals<br>Bread[2]	22 servings/ week	51.0%
**Fish** (servings/ week)	-	≥ median	White fish<br>Shell fish<br>Oily fish	1.5 servings/ week	53.1%
**Beneficial food group for MD in pregnancy but not original MD score**					
**Milk and dairy products** (g/week)	≤ median	≥ median	Milk[3]	6553.3g/week	50.1%
**Deleterious food group**					
**Meat/meat products** (servings/ week)	≤ median	≤ median	Red Meat<br>Sausages/burgers<br>Pies/pasties	5 servings/ week	49.2%

		Poultry<br>Offal
**Food groups from original MD score not included in the MD adapted to pregnancy**		
**Monounsaturated to saturated fat ratio** (servings/ week)	≥ median	-
**Ethanol** (g/day)	Men:<br>10-50 g/day<br>Women:<br>5-25 g/day	-

* 1 point if criterion met; 0 point otherwise

[1] frequency of consumption (i.e. number of servings/week) of these food groups were summed together to obtain an overall 'weekly consumption' for each component of the MD score

[2] Bread consumption was recorded as the number of slices consumed per day. This was multiplied by 7, to obtain weekly consumption.

[3] Milk consumption estimated based on responses to questions asking the mother about how often she consumed milk as a drink on its own, milky drinks and milky puddings. This data was combined with milk consumption through tea, coffee and cereal. As these could not all be readily converted into a frequency of consumption we estimated actual consumption in grams.

Online Table S2. Characteristics of mothers and offspring who were included in analyses and those who were excluded (i.e. mother-child pairs with information on maternal MD score and without information on childhood outcome data) (n=11,993)

	**Included (n=8,907)**	**Excluded (n=3,086)**	***P***
**Maternal Mediterranean diet score**, m (sd)	3.9 (1.5)	3.5 (1.5)	< .001
**Mother's age** (years), m (sd)	28.9 (4.6)	26.5 (5.1)	< .001
**Parity (living children),** %			
0	45.5	43.1	
1	36.0	34.3	< .001
≥2	18.5	22.7	
**Sex of child,** %			
Male	51.2	52.1	0.38
Female	48.8	47.9	
**Multiple pregnancy,** %			
Singleton	97.6	97.2	0.22
Twin	2.4	2.8	
**Season of birth**, %			
Winter	16.1	15.9	
Spring	27.1	26.7	0.56
Summer	30.1	31.4	
Autumn	26.8	26.1	
**Breastfeeding duration**, %			
Never	21.2	35.7	
<3 months	31.6	32.9	< .001
3-6 months	13.8	10.4	
≥6 months	33.5	20.9	
**Mother's educational level,** %			
Certificate of Secondary Education	15.4	32.8	
Vocational	9.0	12.3	< .001
Ordinary level	35.5	32.4	
Advanced level	25.1	15.7	
Degree	15.1	6.9	
**Maternal ethnicity**, %			
White	98.2	95.5	< .001
Non-white	1.8	4.5	
**Housing tenure,** %			
Owned/mortgaged	83.7	62.3	
Council rented	9.4	24.1	< .001
Non-council rented	6.8	13.6	
**Financial difficulties,** %			
Yes	17.1	22.9	< .001

**Maternal history of atopic diseases,** %			
Yes	68.4	68.9	0.63
**Maternal anxiety score in pregnancy,** %			
0-9	21.2	16.9	
10-14	25.7	21.4	< .001
15-20	25.9	24.6	
≥20	27.2	37.2	
**Maximum maternal tobacco exposure,** %			
None	26.5	17.2	
Passive only	45.9	36.0	< .001
1-9 cig/day	7.9	9.6	
10-19 cig/day	11.4	20.0	
20+ cig/day	8.3	17.2	
**Maternal paracetamol use during pregnancy,** %	62.4	64.9	0.01
Yes			
**Maternal antibiotic use during pregnancy,** %			
Yes	16.1	14.6	0.04
**Maternal supplement use during pregnancy,** %			
Yes	56.8	58.9	0.05
**Maternal infections in pregnancy,** %			
Yes	45.8	46.9	0.28
**Total energy intake** (kJ/day), m (sd)	7271 (1962)	7174 (2147)	0.02
**Maternal pre-pregnancy BMI,** %			
<18.50 kg/m²	4.3	6.4	
18.50-24.99 kg/m²	75.3	72.8	< .001
25.00-29.99 kg/m²	15.1	15.0	
≥30.00 kg/m²	5.3	5.9	
**Birth weight,** %			
<2500 g	4.3	5.6	
2500-2999 g	13.8	15.1	0.001
3000-3499 g	35.5	36.6	
3500-3999 g	33.2	30.8	
≥4000 g	13.3	11.9	
**Gestational age** (weeks), m (sd)	39.5 (1.8)	39.4 (1.8)	0.06
**Child's BMI at 7,** %			
<15.00 kg/m²	28.1	32.7	
15.00-17.49 kg/m²	52.5	41.8	0.38
17.50-20.49 kg/m²	15.2	18.2	
≥20.50 kg/m²	4.2	7.3	

**Maternal weight gain during pregnancy**, %			
Quartile 1 (<9.7 kg)	25.3	28.2	
Quartile 2 (9.7 – 12.5 kg)	24.8	24.4	< .001
Quartile 3 (12.5 – 15.5 kg)	25.5	22.1	
Quartile 4 (≥15.5 kg)	24.4	25.3	

Online Table S3. Associations between maternal Mediterranean diet score during pregnancy (binary and continuous) and lung function at 15 years (n=3,549)

	**Mediterranean diet score**			
	**4-7 versus 0-3**	*P*-value	**Per unit increase**	*P*-trend
**FEV_1** (n=3,404)				
β [a] (95% CI)	0.04 (-0.03, 0.11)	0.31	0.01 (-0.01, 0.04)	0.33
β [b] (95% CI)	0.04 (-0.03, 0.12)	0.27	0.02 (-0.01, 0.04)	0.23
**FVC** (n=3,549)				
β [a] (95% CI)	0.02 (-0.06, 0.09)	0.67	0.01 (-0.02, 0.03)	0.51
β [b] (95% CI)	0.02 (-0.05, 0.10)	0.54	0.01 (-0.01, 0.04)	0.32
**FEF_{25-75}** (n=3,549)				
β [a] (95% CI)	0.05 (-0.02, 0.12)	0.20	0.02 (0.00, 0.05)	0.06
β [b] (95% CI)	0.04 (-0.04, 0.11)	0.32	0.02 (0.00, 0.04)	0.10

β: difference in age, height and gender adjusted standard deviation units

[a] Controlling for energy intake

[b] Controlling for energy intake, smoking, infections, supplements, antibiotics and paracetamol use during pregnancy; maternal educational level, housing tenure, financial difficulties, ethnicity, age, parity, maternal history of atopic diseases, anxiety score; sex of child, season of birth, multiple pregnancy, breastfeeding duration

Online Figure S1. Directed acyclic graph showing potential confounders and mediators of the associations between maternal Mediterranean diet score in pregnancy and offspring respiratory and atopic outcomes

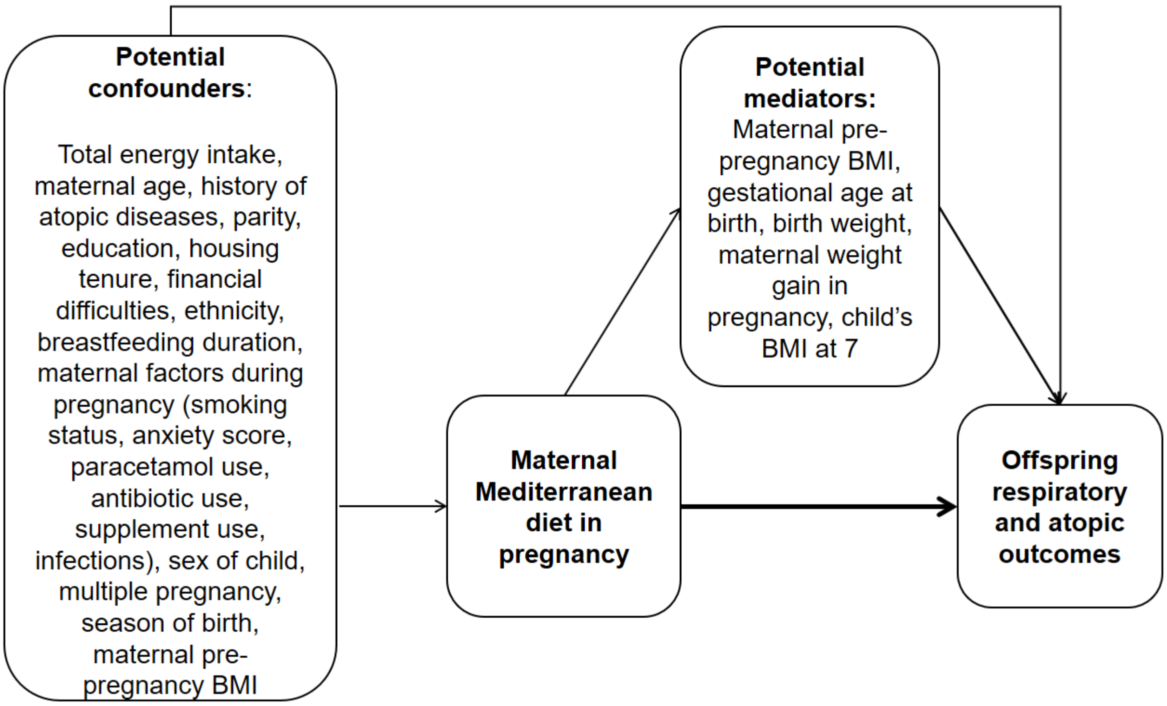

Online Figure S2. Participant flow

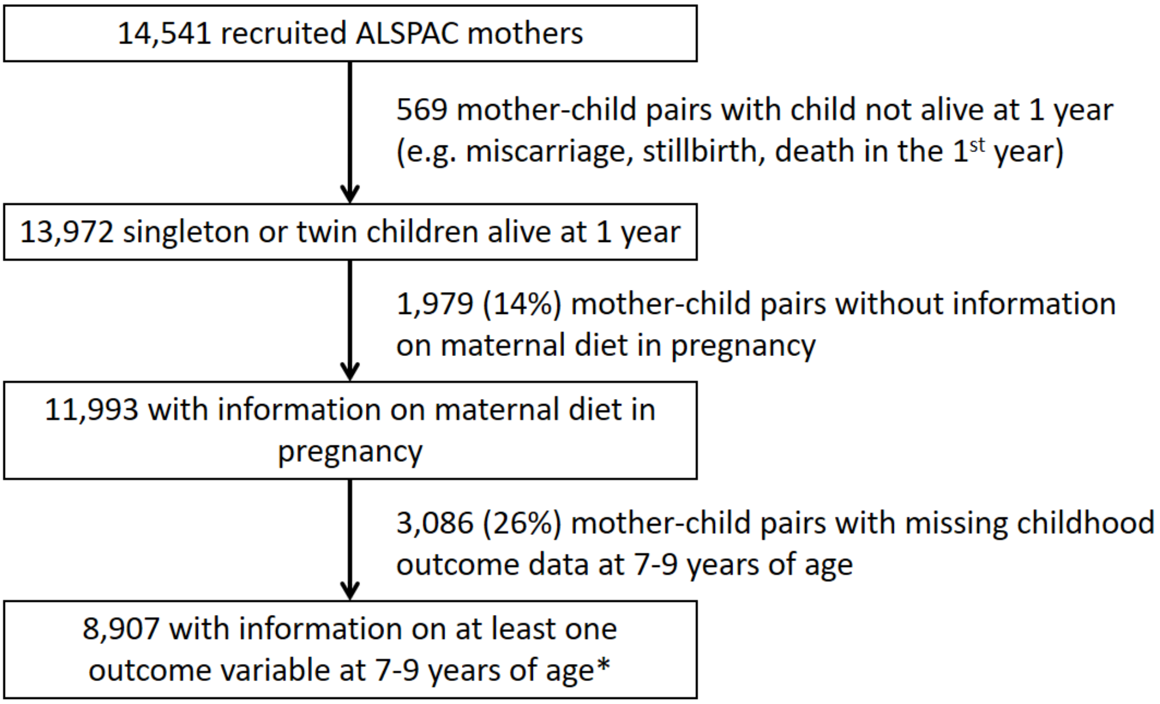

* Once women with information on maternal diet in pregnancy and childhood outcomes at 7-9 years were selected, there was <5% missing data for the covariates, hence we considered the missing information as an additional category for each covariate

References

1. Hernán MA, Hernandez-Diaz S, Robins JM. A structural approach to selection bias. *Epidemiology* 2004; 15: 615–625.
2. Trichopoulou A, Kouris-Blazos A, Wahlqvist ML, Gnardellis C, Lagiou P, Polychronopoulos E, Vassilakou T, Lipworth L, Trichopoulos D. Diet and overall survival in elderly people. *BMJ* [Internet] 1995; 311: 1457–1460Available from: http://www.ncbi.nlm.nih.gov/pubmed/10874552.
3. Chatzi L, Torrent M, Romieu I, Garcia-Esteban R, Ferrer C, Vioque J, Kogevinas M, Sunyer J. Mediterranean diet in pregnancy is protective for wheeze and atopy in childhood. *Thorax* 2008; 63: 507–513.
