# Supplementary material for: Mediterranean diet during pregnancy and childhood respiratory and atopic outcomes: birth cohort study
Source: Eur Respir J. 2020 Mar 12;55(3):1901215. doi: 10.1183/13993003.01215-2019 (PMC7066469; doi:10.1183/13993003.01215-2019)
Supplement: Supplementary file 2 [file ERJ-01215-2019.Shareable.pdf]

ORIGINAL ARTICLE
PAEDIATRICS

SHAREABLE PDF

Mediterranean diet during pregnancy and childhood respiratory and atopic outcomes: birth cohort study

Annabelle Bédard[1], Kate Northstone[2], A. John Henderson[2] and Seif O. Shaheen[1]

Affiliations: [1]Centre for Primary Care and Public Health, Barts and The London School of Medicine and Dentistry, Queen Mary University of London, London, UK. [2]Population Health Sciences, Bristol Medical School, University of Bristol, Bristol, UK.

Correspondence: Seif O. Shaheen, Centre for Primary Care and Public Health, Blizard Institute, Barts and The London School of Medicine and Dentistry, 58 Turner Street, London E1 2AB, UK.
E-mail: s.shaheen@qmul.ac.uk

@ERSpublications
Adherence to a Mediterranean diet during pregnancy is not associated with a reduced risk of asthma or other allergic outcomes in the offspring, but may be associated with increased small airway function in childhood http://bit.ly/382yGcF

Cite this article as: Bédard A, Northstone K, Henderson AJ, *et al*. Mediterranean diet during pregnancy and childhood respiratory and atopic outcomes: birth cohort study. *Eur Respir J* 2020; 55: 1901215 [https://doi.org/10.1183/13993003.01215-2019].

This single-page version can be shared freely online.

ABSTRACT Evidence for associations between Mediterranean diet during pregnancy and childhood asthma, allergy and related outcomes is conflicting. Few cohorts have followed children to school age, and none have considered lung function.

In the Avon Longitudinal Study of Parents and Children, we analysed associations between maternal Mediterranean diet score during pregnancy (estimated by a food frequency questionnaire, using an *a priori* defined score adapted to pregnant women; score ranging from 0 (low adherence) to 7 (high adherence)) and current doctor-diagnosed asthma, wheeze, eczema, hay fever, atopy and lung function in 8907 children at 7–9 years. Interaction between maternal Mediterranean diet and maternal smoking in pregnancy was investigated.

The maternal Mediterranean diet score was not associated with asthma or other allergic outcomes. Weak positive associations were found between maternal Mediterranean diet score and childhood maximal mid-expiratory flow (forced expiratory flow at 25–75% of forced vital capacity ($FEF_{25-75\%}$)) after controlling for confounders. Higher Mediterranean diet scores were associated with increased $FEF_{25-75\%}$ z-scores adjusted for age, height and sex (β 0.06, 95% CI 0.01–0.12; p=0.03, comparing a score of 4–7 *versus* a score of 0–3). Stratifying associations by maternal smoking during pregnancy showed that associations with $FEF_{25-75\%}$ were only seen in children of never-/passive-smoking mothers, but no evidence for a statistically significant interaction was found.

Results suggest adherence to a Mediterranean diet during pregnancy may be associated with increased small airway function in childhood, but we found no evidence for a reduced risk of asthma or other allergic outcomes.

Copyright ©ERS 2020. This article is open access and distributed under the terms of the Creative Commons Attribution Licence 4.0.
